# Early megakaryocyte lineage-committed progenitors in adult mouse bone marrow

**DOI:** 10.1097/BS9.0000000000000187

**Published:** 2024-05-07

**Authors:** Zixian Liu, Jinhong Wang, Yao Ma, Miner Xie, Peng Wu, Sen Zhang, Xiaofang Wang, Fang Dong, Hui Cheng, Ping Zhu, Mingzhe Han, Hideo Ema

**Affiliations:** State Key Laboratory of Experimental Hematology, National Clinical Research Center for Hematological Disorders, Haihe Laboratory of Cell Ecosystem, Institute of Hematology and Blood Diseases Hospital, Chinese Academy of Medical Sciences and Peking Union Medical College, Tianjin, China

**Keywords:** Differentiation, Hematopoietic progenitor cells, Hematopoietic stem cells, Lineage commitment, Megakaryocytes

## Abstract

Hematopoietic stem cells (HSCs) have been considered to progressively lose their self-renewal and differentiation potentials prior to the commitment to each blood lineage. However, recent studies have suggested that megakaryocyte progenitors (MkPs) are generated at the level of HSCs. In this study, we newly identified early megakaryocyte lineage-committed progenitors (MgPs) mainly in CD201^−^CD48^−^ cells and CD48^+^ cells separated from the CD150^+^CD34^−^Kit^+^Sca-1^+^Lin^−^ HSC population of the bone marrow in adult mice. Single-cell colony assay and single-cell transplantation showed that MgPs, unlike platelet-biased HSCs, had little repopulating potential in vivo, but formed larger megakaryocyte colonies in vitro (on average 8 megakaryocytes per colony) than did previously reported MkPs. Single-cell RNA sequencing supported that HSCs give rise to MkPs through MgPs along a Mk differentiation pathway. Single-cell reverse transcription polymerase chain reaction (RT-PCR) analysis showed that MgPs expressed Mk-related genes, but were transcriptionally heterogenous. Clonal culture of HSCs suggested that MgPs are not direct progeny of HSCs. We propose a differentiation model in which HSCs give rise to MgPs which then give rise to MkPs, supporting a classic model in which Mk-lineage commitment takes place at a late stage of differentiation.

## 1. INTRODUCTION

In the classical hierarchy model, hematopoietic stem cells (HSCs) progressively lose their self-renewal and then differentiate into unipotent progenitor cells.^1–3^ Recent studies using cell purification,^4–7^ cell tracking,^8^ and RNA sequencing technologies^9,10^ have provided a different view of hematopoiesis. In particular, the megakaryocyte (Mk) lineage develops in close association with HSCs in the bone marrow of adult mice.

Mk differentiation potential has been detected in CD150^+^CD48^+^Flk2^−^c-Kit^+^Sca-1^+^Lin^−^ (KSL) cells (MPP2),^11^ CD41^+^KSL cells,^12^ and CD150^+^CD41^+^c-Kit^+^Sca-1^−^Lin^−^ cells (MkP).^13^ Single-cell transplantation has revealed myeloid and Mk lineage-restricted repopulating progenitors in CD150^+^CD34^−^KSL cells.^4^ Platelet (Plt)-biased HSCs, marked with Vwf expression, have been reported as a subset of HSCs that give rise to only Mk lineage in primary transplantation but establish multilineage reconstitution in secondary transplantation.^7,8^ These repopulating cells (RCs) seem to play a role under stressed conditions.^7,14^ Moreover, in vivo tracking studies have shown that Mks are an immediate progeny of HSCs in unperturbed hematopoiesis.^8^ Several studies have revealed similar gene expression profiles between HSCs and Mks, suggesting a developmentally close relationship.^6,8,11,15,16^ Some studies have suggested that megakaryocyte progenitors (MkPs) directly arise from HSCs without going through the stage of common myeloid progenitors (CMPs) or Mk/erythrocyte progenitors, both in mice and humans.^12,16,17^

Despite all these studies, the relationship between HSCs and Mk lineage-committed progenitors, for instance, Mk differentiation pathways from HSCs, has not been thoroughly characterized to date. In this study, during a process to increase the purity of HSCs, we encountered new populations significantly enriched in early megakaryocyte lineage-committed progenitors (MgPs) in the mouse bone marrow. These MgPs appeared to be phenotypically close to but functionally distinct from HSCs. Importantly, our identified MgPs showed little repopulating activity in vivo, suggesting that MgPs fundamentally differ from Plt-biased HSCs.

## 2. MATERIALS AND METHODS

### 2.1. Mice

C57BL/6 mice congenic for the Ly5 locus (B6-CD45.1) and C57BL/6 (B6-CD45.2) mice and β-actin-GFP transgenic B6 mice (GPF Tg mice)^18^ were obtained from the animal facility of State Key Laboratory of Experimental Hematology. All the experimental protocols were approved by the Animal Care and Use Committee of the Institute of Hematology and Blood Disease Hospital.

### 2.2. Flow-cytometric sorting

**Figure 1.**
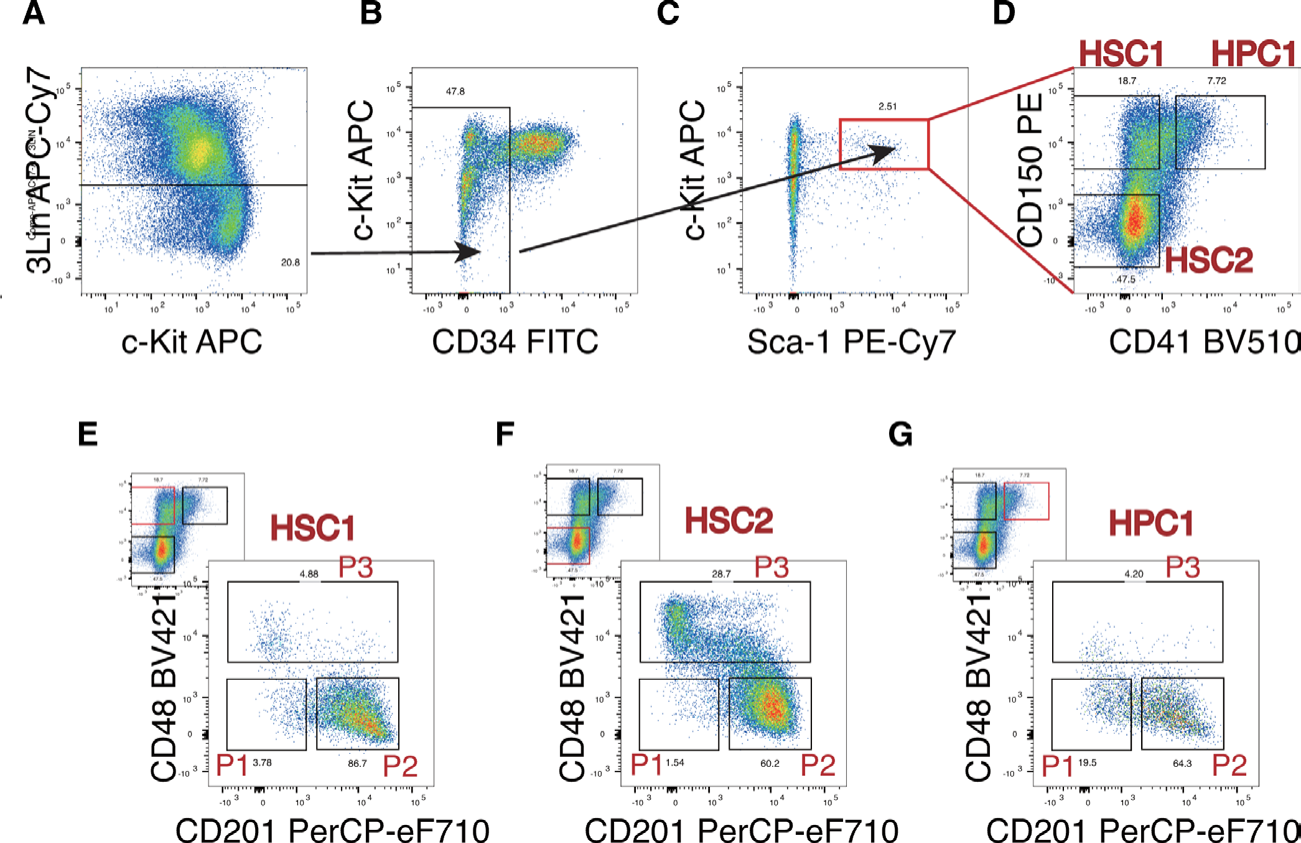
Gating strategy. Bone marrow cells were stained with antibodies against 3Lin, CD34, c-Kit, Sca-1, CD150, CD41, CD48, and CD201 antigens. (A) The gating of 3Lin^−^ cells. (B) The gating of CD34^−^ cells in 3Lin^−^ cells. (C) The gating of c-Kit^+^ and Sca-1^+^ cells in CD34^−^Lin^−^ cells (CD34^−^KSL cells). (D) The gating of CD150^+^CD41^−^ cells (HSC1), CD150^−^CD41^−^ cells (HSC2), and CD150^+^CD41^+^ cells (HPC1) in CD34^−^KSL cells. (E, F, G) The HSC1, HSC2, and HPC1 populations were each divided into 3 fractions (P1, 2, and 3) based on the expression of CD201 and CD48. P1, CD201^−^CD48^−^ cells; P2, CD201^+^CD48^−^ cells; P3, CD48^+^ cells. HSC1-P1/2/3 accounted for 5.2 ± 2.8/7.6 ± 3.9/2.4 ± 0.8 (mean ± SD, n = 5) of CD34^−^KSL cells. HSC2-P1/2/3 accounted for 3.8 ± 3.0/11.4 ± 6.8/26.0 ± 19.4 (n = 5) of CD34^−^KSL cells. HPC1-P1/2/3 accounted for 2.0 ± 0.8/1.9 ± 2.0/1.0 ± 0.5 (n = 5) of CD34^−^KSL cells. HPC = hematopoietic progenitor cell, HSC = hematopoietic stem cell, SD = standard deviation.

Bone marrow cells were flushed from femora, tibiae and pelves of 8- to 10-week-old B6 mice with 2% fetal calf serum (HyClone, Waltham, Massachusetts) in phosphate buffered saline (PBS). The c-Kit^+^ cells were enriched by anti-c-Kit microBeads (catalog no. 130-91-224, Miltenyi Biotech, Germany). Cells were stained with the 3Lin cocktail consisting of APC-efluor (eF) 780-conjugated anti-Ter119 (clone RA3-6B2, catalog no. 47-0452-82, eBioscience, San Diego, California), Gr-1 (clone RB6-8C5, catalog no. 47-5931-82, eBioscience), and B220 (clone RA3-6B2, catalog no.47-0452082, eBioscience) antibodies,^19^ and APC-conjugated anti-c-Kit (clone 2B8, catalog no. 17-1171-82, eBioscience), fluorescein isothiocyanate (FITC)-conjugated anti-CD34 (Clone RAM34, catalog no. 11-0341085, eBioscience), PE/cyanin7 (PE-Cy7) conjugated anti-Sca-1(clone D7, catalog no. 25-5981-82, eBioscience), PE-conjugated anti-CD150 (clone TC15-12F12.2 catalog no. 115904, Biolegend), BV510 conjugated anti-CD41 (clone MWReg 30 catalog no.133923, eBioscience), BV421-conjugated anti-CD48 (clone HM48-1 catalog no. 562745, BD bioscience), and PerCP-eF710 anti-CD201 (clone eBio1560 catalog no. 4338614, eBioscience) antibodies. Analysis and sorting of cells were performed by BD FACSAriaIII flow cytometer. As previously described,^19–23^ the HSC1 population was defined as CD150^+^CD41^−^CD34^−^c-Kit^+^Sca-1^+^Lin^−^ cells; the HSC2 population was defined as CD150^−^CD41^−^CD34^−^c-Kit^+^Sca-1^+^Lin^−^ cells; and the hematopoietic progenitor cell (HPC) 1 population was defined as CD150^+^CD41^+^CD34^−^c-Kit^+^Sca-1^+^Lin^−^ cells (**Fig. 1A–D**). The HSC1, HSC2, and HPC1 (HSC1/HSC2/HPC1) populations were further divided into CD201^−^CD48^−^ cells (P1), CD201^+^CD48^−^ cells (P2); and CD48^+^ cells (P3) (**Fig. 1E–G**).

### 2.3. Single-cell colony assay

Thirty single cells were sorted from P1, 2, and 3 fractions of HSC1, HSC2, and HPC1 populations (HSC1/HSC2/HPC1-P1/2/3), plated in an U-bottom 96-well plate by flow cytometry, and cultured in 200 µL α-minimum essential medium (MEM) supplemented with 10% fetal bovine serum (FBS), 50 U of penicillin/streptomycin, 50 ng/mL murine stem cell factor (SCF), 50 ng/mL murine thrombopoietin (TPO), 10 ng/mL murine IL-3 (IL-3), and 1 IU/mL human erythropoietin (EPO). After 14 days of culture, the diameters of colonies were measured using ImageJ 1.52a software. Cells were morphologically identified as neutrophils (n), macrophages (m), erythroblasts (E), and Mks after Cytospin was prepared for each colony. Mk colonies were identified by morphology under an inverted microscope, some of which were further stained with anti-CD41 antibody. Single-cell colony assay was also performed on CD150^+^CD48^+^Flk2^−^KSL cells (MPP2)^11^ and CD150^+^CD41^+^c-Kit^+^Sca-1^−^Lin^−^ cells (MkP population)^13^ (Supplemental Figure 1, http://links.lww.com/BS/A87). Colonies are defined as those with ≥50 cells in 1 well. Besides, Mk colonies were defined as those with ≥3 cells in 1 well.^24^

### 2.4. Transplantation

Ten cells or single cells from bone marrow cells of B6-CD45.1 mice or GFP Tg mice were sorted and transplanted into lethally irradiated (9.5 Gy) B6-CD45.2 mice through the tail vein with 5 × 10^5^ bone marrow cells from B6-CD45.2 mice. In 10-cell transplantation, 6 months after the first transplantation, 2 × 10^7^ bone marrow cells were transplanted into 10 lethally irradiated B6-CD45.2 mice. To detect the repopulating activity in progenitor cells, 500 cells isolated from GFP Tg mice were transplanted into sublethally irradiated B6 mice (4.5 Gy) without competitor cells.^13^

### 2.5. Peripheral blood analysis

Peripheral blood of the recipient mice was analyzed by BD FACS Canto II for the myeloid, B cell, and T cell lineage contribution of donor-derived cells as described.^25^ Successful reconstitution was defined as >0.05% CD45.1^+^ or GFP^+^ cells in each lineage and > 0.01% GFP^+^ cells for CD41^+^ Plt at any time after transplantation.

### 2.6. Single-cell RNA sequencing (scRNA-seq)

Single HSC1/HSC2-P1/2/3 cells, MPP2 cells, and MkP cells were directly sorted into 0.2 mL PCR tubes with lysis buffer. The scRNA-seq library was constructed with Smart-seq 2 (illumina) as previously reported.^26,27^ DNA sequencing was performed on an Illumina HiSeq 4000.

### 2.7. Analysis of scRNA-seq data

After reads of adaptor contaminants and low-quality bases were removed, the reads were aligned to the mouse genome (GENCODE M16) using the STAR tool.^28^ The HTSeq package was used to count unique molecular identifiers (UMIs) as the transcript copy number.^29^ A total of 800 highly variable genes were used for the principal component analysis. Uniform manifold approximation and projection (UMAP) plot^30^ was drawn using *RunUMAP* with dims set to 1:13. Monocle R package5 (v.2.18.0) was used to construct the trajectory tree of groups of cells, suggesting the differentiation orders.^31^ We used the Seurat FindAllMarkers to identify unique cluster-specific marker genes.^32^ Gene ontology analysis was performed using DAVID (Bioinformatics Resources 6.8).^33^

### 2.8. Single-cell reverse transcription polymerase chain reaction (single-cell RT-PCR)

Single cells from HSC1/HSC2/HPC1-P1/2/3 were sorted into PCR tubes containing 10 μL reverse transcription and specific-target amplification mixture. RT-PCR was performed by Fluidigm Biomark as described.^25^ The sets of PCR primers, as listed in Supplemental Table 1, http://links.lww.com/BS/A93, were purchased from Thermo Fisher Scientific, Waltham, Massachusetts.

### 2.9. Terminology

Long-term (LT)-HSCs were defined as cells with >6 months myeloid reconstitution potential; and short-term (ST)-HSCs were defined as cells with <6 months myeloid reconstitution potential and B and T lymphoid reconstitution at any time after transplantation, at the clonal level.^19,22,25,34^ MgPs were defined as Mk-lineage-committed functional cells at the clonal level. MkP has been defined as the phenotypically defined population.^13^ Functional cells were designated as MkP cells to be distinguished from the MkP population.

### 2.10. Statistical analyses

One-way analysis of variance (ANOVA), Chi-square tests, Fisher exact test, and unpaired *t* test with Welch correction were performed with statistics 23 (IBM, Armonk, New York). Wilcoxon test was performed with ggpubr R package (v.0.4.0). Significance was indicated as follows: *P < .05; **P < .01; ***P < .001; ****P < .0001.

## 3. RESULTS

### 3.1. Subpopulations in HSC1, HSC2, and HPC1

CD150^+^CD41^−^CD34^−^c-Kit^+^Sca-1^+^Lin^−^ (HSC1) cells are enriched in myeloid-biased HSCs, whereas CD150^−^CD41^−^CD34^−^c-Kit^+^Sca-1^+^Lin^−^ cells (HSC2) are enriched in lymphoid-biased HSCs.^4,21,22,25,35,36^ CD150^+^CD41^+^CD34^−^c-Kit^+^Sca-1^+^Lin^−^ (HPC1) cells are enriched in myeloid-biased HPCs.^4,37^ Based on the expression of CD201 and CD48, the HSC1, HSC2, and HPC1 (HSC1/HSC2/HPC1) populations, respectively, were further divided into CD201^−^CD48^−^ (P1), CD201^+^CD48^−^ (P2), and CD48^+^ (P3) populations (**Fig. 1**). The HSC1-P2 has been reported to be further enriched in myeloid-biased HSCs,^25,34–36^ but the other populations have not been characterized.

### 3.2. Single-cell colony assay of P1, P2, P3 cells

**Figure 2.**
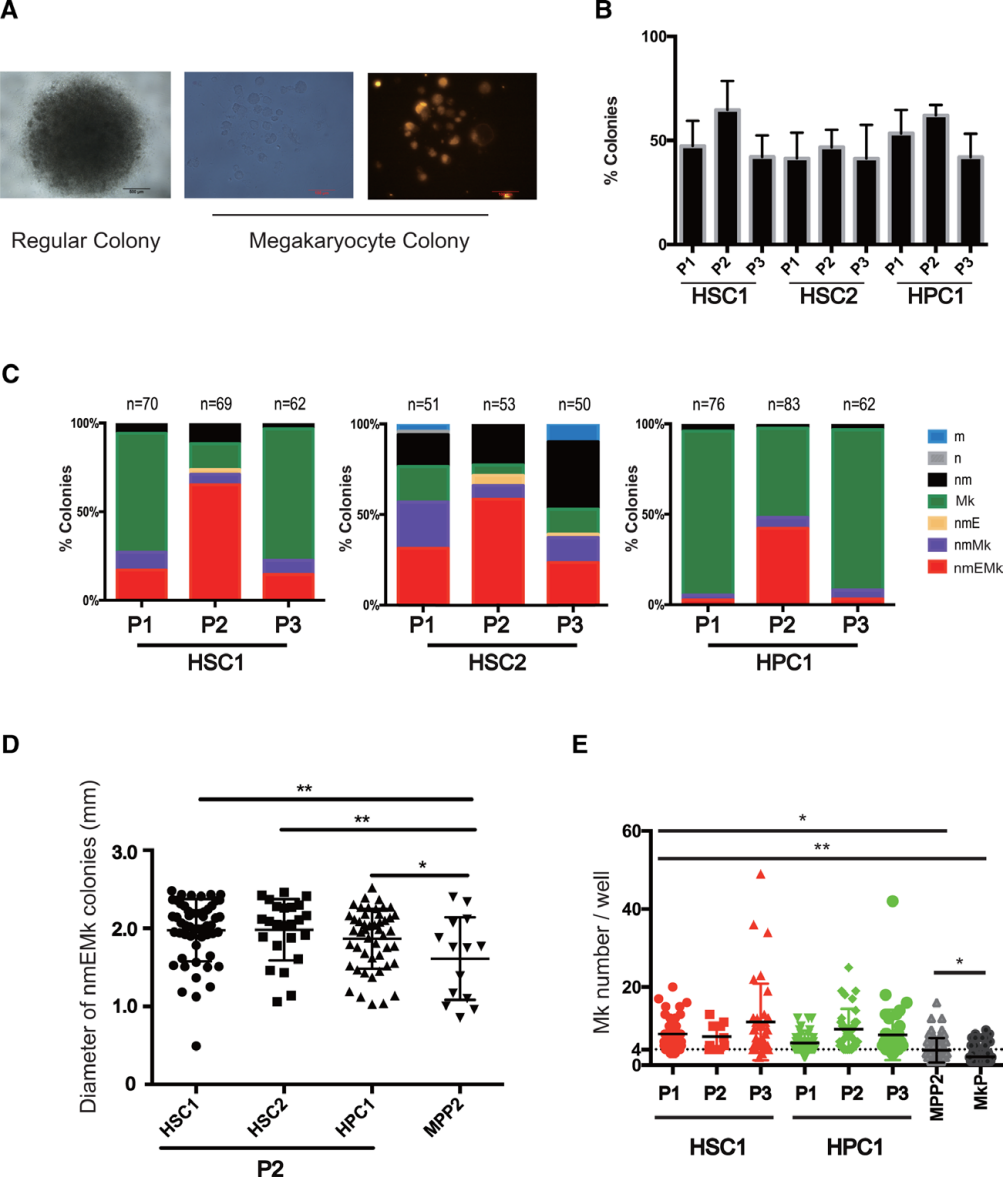
Single-cell colony assays. (A) Two types of colonies on day 14 of culture. The left panel shows a regular colony. The middle panel shows an Mk colony. The right panel shows the CD41 expression in the Mk colony in the middle panel. (B) The frequencies of colony-forming cells. (C) Colony types. The frequency of nmEMk colony-forming cells in HSC1-P2 cells was significantly greater than that in HSC1-P1 cells or HSC1-P3 cells (65.2% vs 17.1% or 14.5%, *P* < .001); that in HSC2-P2 cells than that in HSC2-P1 or HSC2-P3 cells (58.5% vs 31.4% or 24%, *P* < .01); that in HPC1-P2 cells than that in HPC1-P1 or HPC1-P3 cells (42.2% vs 2.6% or 3.2%, *P* < .001) by Chi-square test. The frequency of Mk colony-forming cells in HSC1-P1 or HSC1-P3 cells was significantly greater than that in HSC1-P2 cells (67.1% or 74.2% vs 14.5%, *P* < .001); that in HPC1-P1 or -P3 cells than those in HPC1-P2 cells (90.8% or 88.7 vs 49.4%, *P* < .001) by Chi-square test. (D) Day 14 nmEMk colony size shown as the diameters of colonies. nmEMk colonies formed by HSC1/HSC2/HPC1-P2 cells were significantly larger than those formed by MPP2 cells (*P* = .003, *P* = .008, and *P* = .042, respectively, 1-way ANOVA test). (E) Day 14 Mk colony size. A significant proportion of MPP2 and MkP cells did not divide, but matured into Mks. The numbers of Mks per colony were 8.0 ± 6.5 for HSC1/HPC1-P1/3 cells (mean ± SD, n = 167), 3.8 ± 3.2 for MPP2 cells (n = 58), and 2.2 ± 2.0 for MkP cells (n = 87). The number of Mks per colony of MPP2 cells was significantly smaller than those of HSC1-P1/2/3 cells (*P* < .001, *P* = .038, and *P* < .001) and HPC1-P1/2/3 cells (*P* = .047, *P* < .001, and *P* < .001) by 1-way ANOVA test. The number of Mks per colony of MkP cells was significantly smaller than those of HSC1-P1/2/3 cells (*P* < .001, *P* = .002, and *P* < .001) and HPC1-P1/2/3 cells (*P* < .001 each) by 1-way ANOVA test. The number of Mks per colony of MkP cells was significantly smaller than that of MPP2 cells (*P* = .044; 1-way ANOVA test). *P < .05; **P < .01. ANOVA = analysis of variance, E = erythroblast, HPC = hematopoietic progenitor cell, HSC = hematopoietic stem cell, m = macrophage, Mk = megakaryocyte, MkPs = megakaryocyte progenitors, n = neutrophil, SD = standard deviation.

To compare the HSC1/HSC2/HPC1-P1/2/3 populations, we performed single-cell colony assay. Two kinds of colonies were observed. One was regular colonies (**Fig. 2A**, left panel). The other was Mk colonies comprising a small number of large cells expressing CD41 (**Fig. 2A**, middle and right panels). Colony cells were morphologically classified into neutrophils (n), macrophages (m), erythroblasts (E), and Mks (Supplemental Figure 2A, http://links.lww.com/BS/A88). Multilineage (nmEMk, nmMk, and nmE), bipotent (nm) unipotent (n, m, and Mk) colonies were detected whereas neither EMk nor E colonies were detected (Supplemental Figure 2B http://links.lww.com/BS/A88). The colony-forming efficiencies were similar among the HSC1/HSC2/HPC1-P1/2/3 populations (**Fig. 2B**).

The distribution of nmEMk colonies among the P1/2/3 populations was similar among the HSC1/HSC2/HPC1 populations: the highest frequency of nmEMk colonies was found in P2, suggesting that the enrichment of multipotent cells in P2 in common with the HSC1/HSC2/HPC1 populations (**Fig. 2C**).

There was no significant difference in the diameters among nmEMk colonies derived from the HSC1/HSC2/HPC1-P2 populations (**Fig. 2D**). MPP2 cells also formed nmEMk colonies (Supplemental Figure 3A, http://links.lww.com/BS/A89). The diameters of nmEMk colonies derived from the HSC1/HSC2/HPC1-P2 populations were significantly greater than those from the MPP2 population (**Fig. 2D**).

Mk colonies were formed by all HSC1/HSC2/HSC3-P1/2/3 populations. Most interestingly, a large proportion (73.3 ± 31.3%, n = 5) of all these Mk colonies was formed by the HSC1/HPC1-P1/3 populations (**Fig. 2C**). No difference was seen in the numbers of Mks per colony among the HSC1/HPC1-P1/3 populations. We also detected Mk colony formation by single MPP2 cells (CD150^+^CD48^+^Flk2^−^KSL cells) and MkP cells (CD150^+^CD41^+^c-Kit^+^Sca-1^−^Lin^−^ cells) (Supplemental Figure 3A, http://links.lww.com/BS/A89). The numbers of Mks per colony (8.0 ± 6.5, n = 167) in the HSC1/HPC1-P1/3 populations together were significantly greater than those in the MPP2 and MkP populations (**Fig. 2E**). A large proportion of MkP cells generated only 1 to 3 Mks (Supplemental Figure 3A, http://links.lww.com/BS/A89), consistent with previous studies.^8,13^ The number of Mks per colony in the MPP2 population was significantly greater than that in the MkP population (**Fig. 2E**).

On day 14, some colonies were too small for Cytospin preparation for morphological analysis. To address this problem, we extended the culture period to 3 weeks. Some small colonies became larger by day 21 (Supplemental Figure 3B, http://links.lww.com/BS/A89). The lineage output of those slowly growing colonies appeared to be consistent with that of day 14 colonies. The percentages of nmEMk colonies in the HSC1/HSC2/HPC1-P2 populations were significantly greater than those in the HSC1/HSC2/HPC1-P1/3 populations. The percentages of Mk colonies in the HSC1/HPC1-P1/3 populations were significantly greater than those in the HSC1/HPC1-P2 populations.

In order to examine the relationship between nmEMk and Mk colony-forming cells, we studied myeloid lineage commitment in early divisions of HSCs. When single HSC1-P2 gave rise to 3 to 8 cells in culture, individual cells were isolated by micromanipulation and their colony-forming potential was examined. As shown in Supplemental Table 2, http://links.lww.com/BS/A93, a total of 224 cells derived from 43 single cells were analyzed. Greater than 50% of the cells formed nmEMk colonies, showing the amplification of nmEMk colony-forming cells in early divisions. Greater than 20% of the cells formed nm or m (nm/m) colonies, suggesting EMk or nEMk differentiation potential was lost after 2 or 3 division. Among them, however, Mk colonies were extremely rare. These data suggested that cells with nmEMk differentiation potential can be committed to nm/m lineage from the first division, but the commitment to Mk lineage may require 4 or more divisions.

### 3.3. Little reconstitution by P1 and P3 cells

We performed transplantation with 10 P1, P2, and P3 cells each from the HSC1, HSC2, and HPC1 populations (HSC1/HSC2/HPC1-P1/2/3 cells). Supplemental Figure 4, http://links.lww.com/BS/A90, shows the results of primary and secondary transplantation. As predicted, HSC1-P2 cells were significantly enriched in the LT (>6 months) reconstitution potential while HSC2-P2 cells were significantly enriched in ST (<6 months) reconstitution potential, where LT but not ST reconstitution potential was transplantable to secondary recipients, consistent with previous studies.^4,25,35,37^ Notably, a low level of ST reconstitution was detected in HSC1/HSC2-P1 cells but no such activity was detected in HSC1/HSC2-P3 cells. HPC1-P1/2/3 showed very little reconstitution activity.

**Figure 3.**
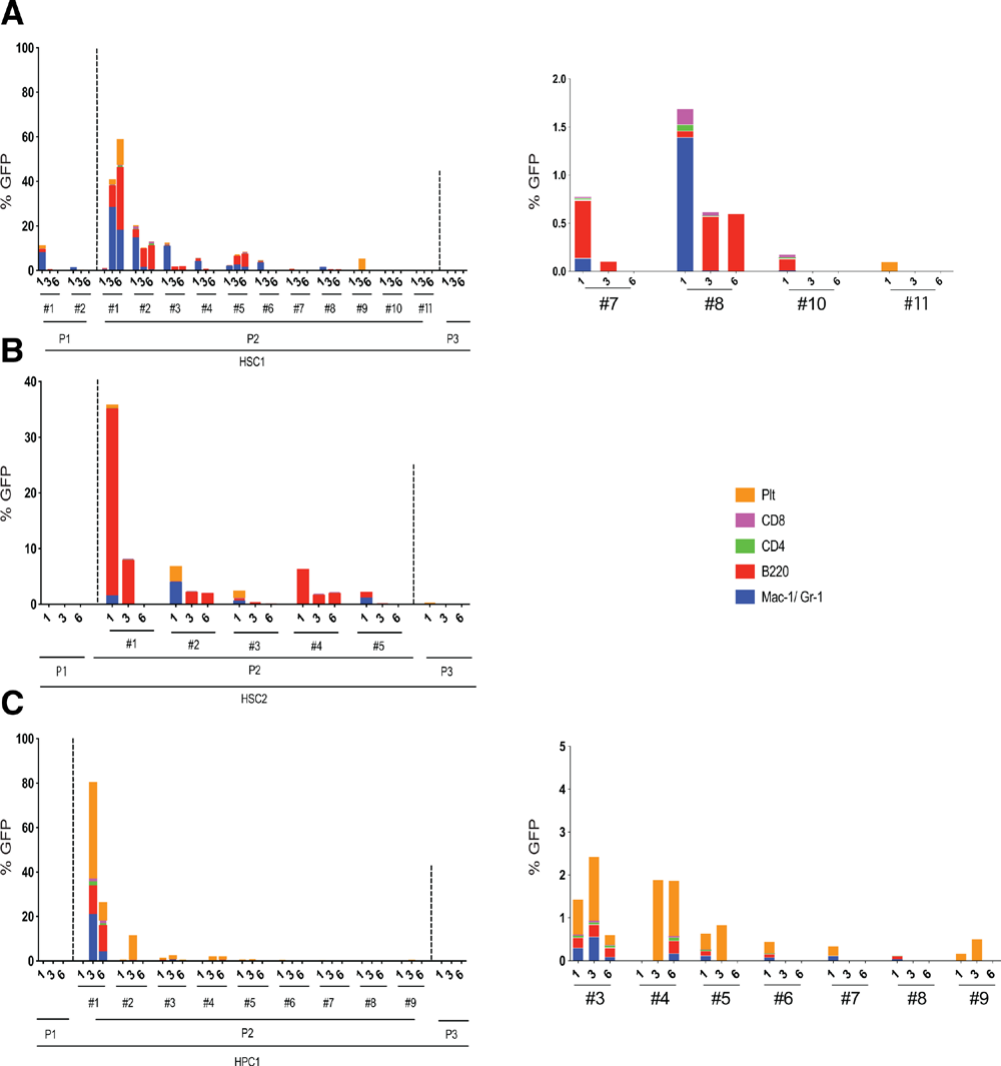
Single-cell transplantation. Single cells were isolated from (A) HSC1-P1/2/3, (B) HSC2-P1/2/3, and (C) HPC1-P1/2/3 populations, mixed with 5 × 10^5^ competitor cells, and injected into 30 lethally irradiated mice. Recipients were analyzed 6 mo after transplantation. Data of reconstituted mice are selectively shown (mouse ID is shown by # number). Each column represents the percentage of total chimerism composed of myeloid, B cell, CD4 T cell, CD8 T cell, and Plt lineages. Data of mice with low chimerism are shown again on the right side with enlargement (A, C). (A) Nineteen recipients of HSC1-P1 cells, 23 recipients of HSC1-P2 cells, and 20 recipients of HSC1-P3 cells survived. (B) Nineteen recipients of HSC2-P1 cells, 16 recipients of HSC2-P2 cells, and 12 recipients of HSC2-P3 cells survived. (C) Fourteen recipients of HPC1-P1 cells, 18 recipients of HPC1-P2 cells, and 14 recipients of HPC1-P3 cells survived. % GFP^+^ leukocytes and % GFP^+^ Plts are together shown in a total of 200% (100% leukocytes and 100% Plts). HPC = hematopoietic progenitor cell, HSC = hematopoietic stem cell, Plt = platelet.

We also performed single-cell transplantation using GFP transgenic mice as donors to detect Plt reconstitution as well (**Fig. 3**). Consistent with bulk cell transplantation, 3 LT-HSCs, 5 ST-HSCs, and 3 lineage-restricted RCs (B cell/Plt, B cell, and Plt-RCs) were detected in HSC1-P2 cells (11/23 mice); 5 ST-HSCs were detected in HSC2-P2 cells (5/16 mice); and 3 LT-HSCs, 4 ST-HSCs, and 2 lineage-restricted RCs (Myeloid and Plt) were detected in HPC1-P2 cells (9/18 mice).

Two ST-HSCs were detected in HSC1-P1 cells (2/19 mice). No reconstitution was found in recipients that received HSC2-P1 or HPC1-P1 cells. One Plt-RC was found in HSC2-P3 cells. No reconstitution was found in mice that received HSC1-P3 or HPC1-P3 cells. Taken together, no significant level of reconstitution potential was detected in single HSC1/HSC2/HPC1-P1/3 cells. Plt-RCs were detected in HSC1-P2, HSC2-P3, and HPC1-P2 cells at a low frequency.

### 3.4. Plt reconstitution potential in P1/2/3 was greater than that of MPP2 and MkP

**Figure 4.**
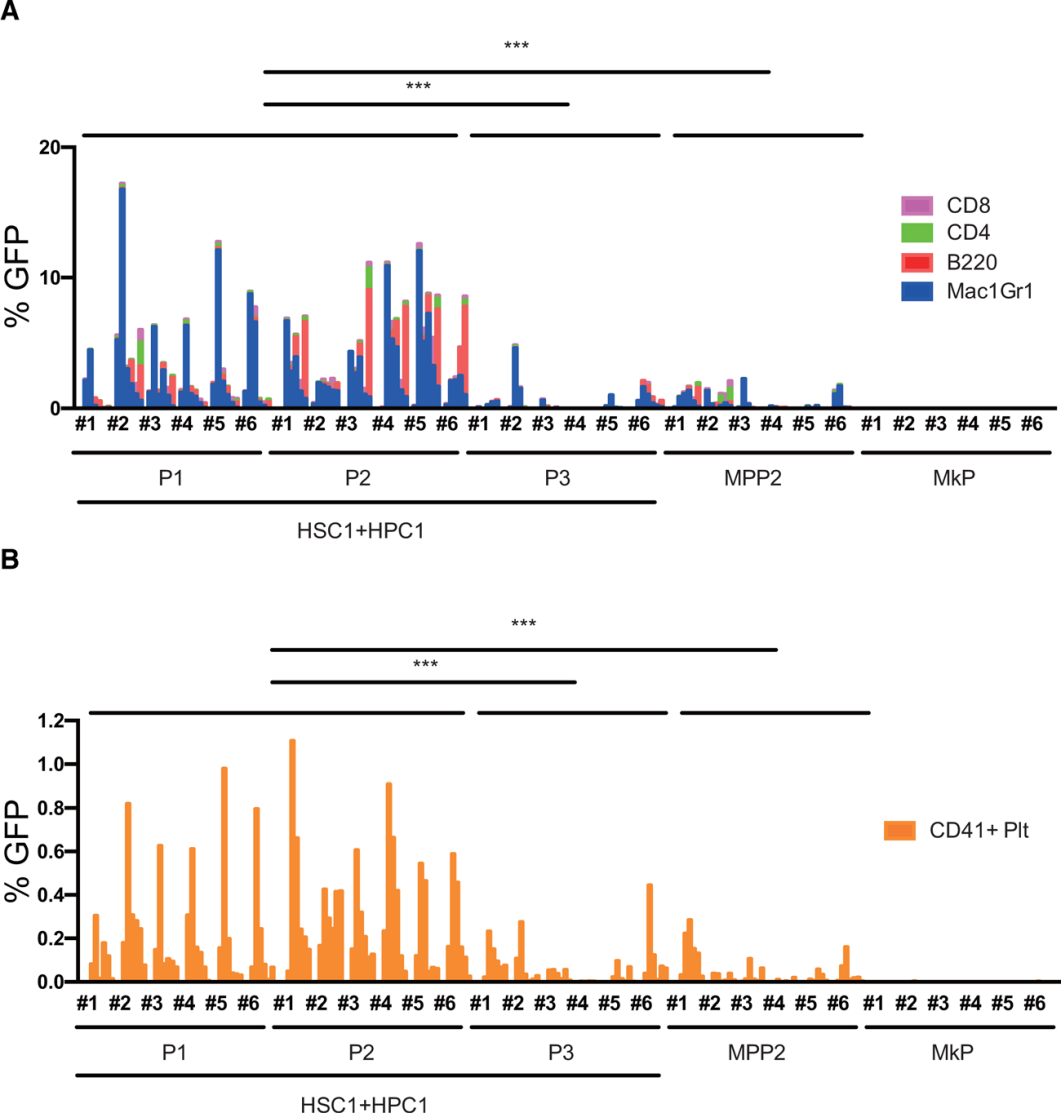
Plt reconstitution by 500 cells. (HSC1 + HPC1)-P1, P2, and P3 cells, MPP2 cells, and MkP cells were isolated from GFP Tg mice and transplanted into sublethally irradiated mice (500 cells per mouse); data of individual mice (#1–6) are shown. PB was analyzed 2–8 wk after transplantation. In this transplantation, CD150^+^CD48^+^Flk2^−^CD34^+^KSL MPP2 cells were used. (A) The percentages of GFP^+^ cells in Gr-1/Mac-1^+^ cells, B220^+^ cells, CD4^+^ cells, and CD8^+^ cells are shown. (B) The percentages of GFP^+^ cells in CD41^+^ platelets are shown. The overall reconstitution activity of P1 or P2 cells was significantly greater than that of MPP2 or P3 in myeolymphoid and Plt lineages (Dunn multiple comparison test). ***P < .001. HPC = hematopoietic progenitor cell, HSC = hematopoietic stem cell, MkPs = megakaryocyte progenitors, Plt = platelet.

It is difficult to detect in vivo HPC activity at the single-cell level. To compare overall Plt reconstitution activity, we transplanted 500 cells each from the (HSC1+HPC1)-P1/2/3 (CD41 was not used to separate the HSC1 from the HPC1 population), MPP2, and MkP populations of GFP transgenic mice into sublethally irradiated mice without competitor cells (**Fig. 4**). ST myeloid and lymphoid reconstitution was detected after transplantation with P1 and P2 cells. A very low level of such an activity was detected after transplantation with P3 and MPP2 cells, but no such activity was detected after transplantation with MkP cells.

Plt reconstitution was also detected after transplantation with P1/2/3 and MPP2 cells, but not with MkPs. These data suggested that the Plt reconstitution activity in P1/2/3 or MPP2 is significantly greater than that in MkP cells and that the Plt reconstitution activity in P1 or P2 cells is significantly greater than that in P3 or MPP2 cells.

### 3.5. Relationship among HSCs and HPCs predicted by single-cell RNA-seq data

**Figure 5.**
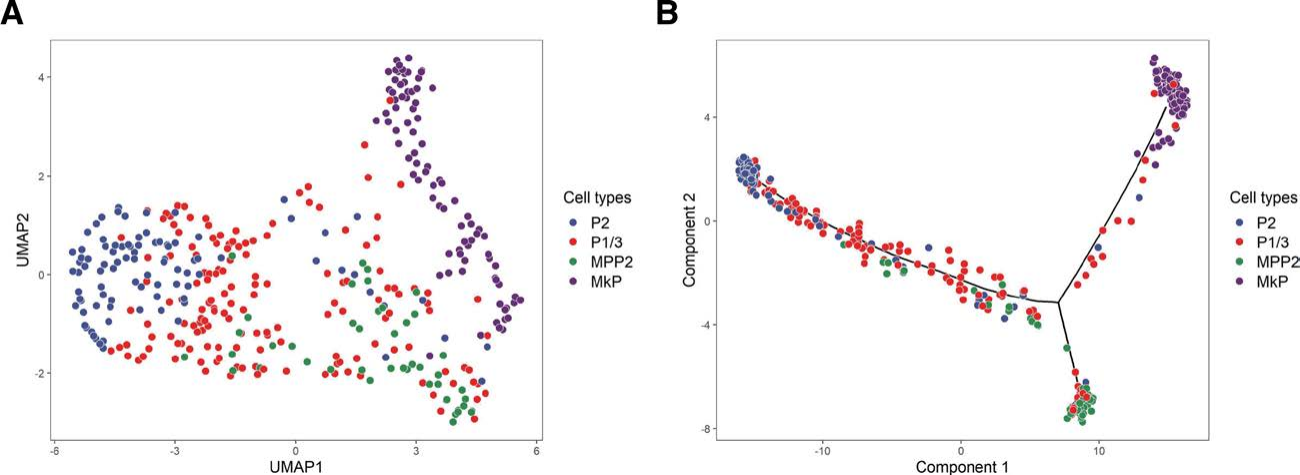
Cluster and trajectory analyses of single-cell RNA-Seq data. (A) UMAP plots of clusters. (B) Monocle trajectory. HSC1/HPC1-P2 cells are shown as P2. HSC1/HPC1-P1/3 cells are shown as P1/3. MPP2 and MkP cells were also analyzed. Data were obtained from analysis of 96 P2 cells, 180 P1/3, 48 MPP2 cells, and 85 MkP cells. HPC = hematopoietic progenitor cell, HSC = hematopoietic stem cell, MkPs = megakaryocyte progenitors, UMAP = uniform manifold approximation and projection.

To deduce their relationship, single-cell RNA sequencing (scRNA-seq) was successfully performed on 48 HSC1-P1 cells, 48 HSC1-P2 cells, 44 HSC1-P3 cells, 44 HPC1-P1 cells, 48 HPC1-P2 cells, 44 HPC1-P3 cells, 48 MPP2 cells, and 85 MkP cells. Colony assay and transplantation data suggested that the HSC1-P2 population was closely related with the HPC1-P2 population so that these populations were analyzed together, and that the HSC1-P1/3 populations were closely related with the HPC1-P1/3 populations so that these populations were analyzed together, as shown in Figure 5.

Cluster analysis showed that HSC1/HPC1-P2 and MkP cells, respectively, form distinct clusters. In contrast, HSC1/HPC1-P1/3 and MPP2 cells were heterogeneous populations and widely distributed between HSC1/HPC1-P2 and MkP clusters (**Fig. 5A**). Trajectory analysis suggested that HSC1/HPC1-P1/3 cells continuously go to the direction of MkP. Interestingly, MPP2 cells shared the common pathway with HSC1/HPC1-P1/3 cells until a certain point and then changed the direction (**Fig. 5B**). These data suggested that HSC1/HPC1-P1/3 cells are downstream of HSC1/HPC1-P2 upstream of MkP cells along the differentiation, and MPP2 cells partially share the differentiation pathway with HSC1/HPC1-P1/3 cells.

We analyzed differentially expressed genes in the HSC1/HPC1-P2, HSC1/HPC1-P1/3, and MkP populations. The data are representatively shown in Supplemental Figure 5, http://links.lww.com/BS/A91. The expression of *Vwf*, *Pl4*, *Gata1*, and *Runx1* in the MkP population was significantly greater than that in the HSC1/HPC1-P2 and -P1/3 populations. The expression of *Procr*, *Sh2n3*, and *Mecom* in the HSC1/HPC1-P2 populations was significantly greater than that in the HSC1/HPC1-P1/3 and MkP populations. The expression of *Spi1* in the HSC1/HPC1-P2 or HSC1/HPC1-P1/3 populations was significantly greater than the MkP population. The expression levels of many genes in the HSC1/HPC1-P1/3 populations appeared to be intermediate between those of the HSC1/HPC1-P2 and MkP populations.

### 3.6. Mk gene expression analysis by single-cell RT-PCR

In general, single-cell RT-PCR is more sensitive than single-cell RNA-seq. Focusing on Mk- and HSC-related genes, the expression of 48 genes was selectively examined with 24 single cells each from HSC1/HSC2/HPC1-P1/2/3. Data are shown as a heatmap format in Supplemental Figure 6, http://links.lww.com/BS/A92.

**Figure 6.**
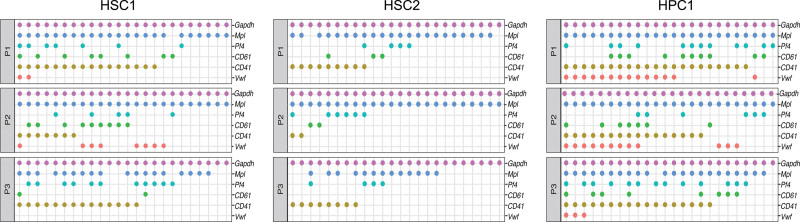
scRT-PCR of *Vwf, CD41, CD61, Pf4* and *Mpl* genes. Each horizontal row represents 24 single cells. Positive cells defined as cells with a threshold cycle value (Ct) < 27.65 are shown as dots. *CD41*^+^*Vwf* ^+^ cells in HPC1-P1/2 (22/48 cells) were significantly more than those in the other populations (6/168) (*P* < .0001). *CD41*^+^/*Pf4*^+^ cells in HSC1-P1/3 and HPC1-P1/3 (33/96 cells) were significantly more than those in the other populations (7/120) (*P* < .0001) (Fisher exact test). HPC = hematopoietic progenitor cell, HSC = hematopoietic stem cell.

We detected Mk lineage markers (*CD41*, *CD61*, *Vwf*, and *Pf4*) as graphically shown in Figure 6. The expression of *CD41* was detected in all HSC1/HSC2/HPC1-P1/2/3 populations although the frequencies of positive cells differed. *CD41*^+^*CD61*^+^ cells were detected in the HPC1-P1/2/3 populations (19/72 cells) slightly more than in the HSC1-P1/2/3 populations (10/ 72 cells). Very few *CD41*^+^*CD61*^+^ cells were detected in the HSC2-P1/2/3 populations. *Vwf* ^+^ cells were detected in the HSC1-P2 and HPC1-P1/2 populations, whereas no *Vwf* ^+^ cells were detected in the HSC2-P1/2/3 and HSC1-P3 populations. A few *Vwf* ^+^ cells were detected in the HSC1-P1 and HPC1-P3 populations. The HPC1-P1/2 populations were significantly enriched in *CD41*^+^*Vwf* ^+^ cells, suggesting that the coexpression of *CD41/Vwf* may mark a certain stage of megakaryopoiesis. The HSC1/HPC1-P1/3 populations were significantly enriched in *CD41*^+^/*Pf4*^+^ cells, suggesting that the coexpression of *CD41/Pf4* is to some extent related to the Mk progenitor activity. Together with scRNA-seq data, these data showed that HSC1/HPC1-P1/3 cells were transcriptionally heterogenous.

## 4. DISCUSSION

We operationally named the phenotypically defined population, CD150^+^CD41^−^CD34^−^KSL cells as HSC1, the phenotypically defined population, CD150^−^CD41^−^CD34^−^KSL cells as HSC2, and the phenotypically defined population, CD150^+^CD41^+^CD34^−^KSL cells as HPC1, respectively. The purification levels of HSC1 and HSC2 significantly increased by using CD201^+^CD150^+^CD48^−^CD41^−^CD34^−^KSL cells (HSC1-P2) and CD201^+^CD150^−^CD48^−^CD41^−^CD34^−^KSL cells (HSC2-P2), respectively (**Fig. 3**, Supplemental Figure 4, http://links.lww.com/BS/A90). The HSC1-P2 population is enriched in myeloid-biased LT-HSCs whereas the HSC2-P2 population is enriched in lymphoid-biased ST-HSCs. These HSCs may play different roles in hematopoiesis. For example, lymphoid-biased ST-HSCs may contribute to lymphoid development at the embryonic stage, and myeloid-biased LT-HSC may contribute to the maintenance of life-long myelopoiesis.

The expression level of CD41 only distinguished the HPC1 or HPC1-P2 population from the HSC1 or HSC1-P2 population. Transplantation data of single HPC1-P2 cells appeared to be inconsistent with those of 10 HPC1-P2 cells (**Fig. 3**, Supplemental Figure 4, http://links.lww.com/BS/A90). It seemed to be sometimes difficult to discriminate HSC1 cells from HPC1 cells by anti-CD41 antibody staining as previously reported.^19^

The HSC1/HSC2-P2 populations were enriched in multilineage, particularly nmEMk colony-forming cells (**Figs. 2, 3** and Supplemental Figure 3, http://links.lww.com/BS/A89, Supplemental Figure 4, http://links.lww.com/BS/A90). These data suggested that ST- and LT-HSCs form multilineage colonies in vitro. The HSC1-P1/3 and HPC1-P1/3 populations were significantly enriched in Mk colony-forming cells although they barely repopulated in vivo (**Figs. 2, 3** and Supplemental Figure 3, http://links.lww.com/BS/A89, Supplemental Figure 4, http://links.lww.com/BS/A90). We named these early Mk-lineage-committed progenitors MgPs. MgPs did not phenotypically overlap MkPs at all (Supplemental Table 3, http://links.lww.com/BS/A93). Unlike the phenotypically defined CMP, MPP, and MkP populations, MgPs were functionally defined by single-cell colony assay. MgPs were rather widely distributed in four or more populations (Supplemental Table 3, http://links.lww.com/BS/A93). MgPs may partially overlap pMKPs which have been reported to be expanded in the mouse essential thrombocythemia model.^38^ MgPs appeared to be more immature than MkPs^11,13^ because MgPs generated on average 8 cells per cell, significantly more Mks than did MkPs in vitro, also supported by in vivo Plt reconstitution activity (**Figs. 2, 4**). To our knowledge, MgPs are the earliest Mk progenitors in the bone marrow of adult mice. Since nmEMk colony-forming cells and MgPs were detected in the same populations (**Fig. 2**, Supplemental Figure 3, http://links.lww.com/BS/A89), these cells should be developmentally related. We assumed that both functionally defined HSCs and CMPs have the nmEMk differentiation potential. It is difficult to separate CMPs from HSCs mostly because the in vivo activity of CMPs is too low to be detected at the single-cell level.

To illustrate the relationship between MgPs and HSCs, we performed in vitro clonal analysis of 2 to 8 cells generated from single HSCs in culture (Supplemental Table 2, http://links.lww.com/BS/A93). The loss of EM differentiation potential likely took place as early as from the first division. It was assumed that 4 or more divisions are required for HSCs to generate MgPs. These data suggested that MgPs are not immediate progeny of HSCs.

Plt-RCs were detected in the HSC1-P2, HSC2-P2/3, and HPC1-P2 populations at very low frequencies (**Fig. 3**). The activity of Plt-RCs seemed to be lower, and their lifespan seemed to be shorter than those of Plt-biased HSCs.^7,8^ Since we used 5 × 10^5^ instead of 2 × 10^5^ bone marrow cells as competitive cells, our transplantation assay might not be sensitive enough to detect Plt-biased HSCs with robust and stable Pltv reconstitution potential. Alternatively, the frequency of Plt-biased HSCs in wild type mice might be much lower than that in Vwf-tdTomato transgenic mice.^7–9^

**Figure 7.**
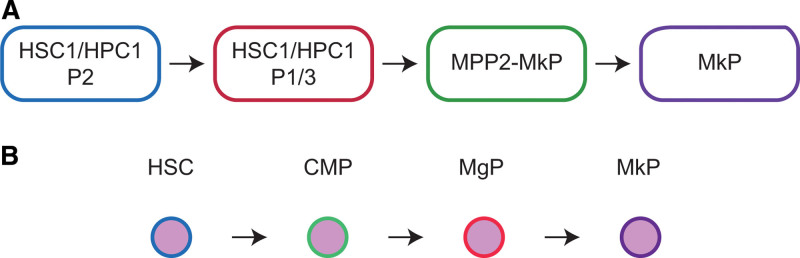
MgPs give rise to MkPs. (A) Hierarchical relationship among HSC and HPC populations. The HSC1/HPC1-P2 populations give rise to the HSC1/HPC1-P1/3 populations which give rise to the MkP population through MPP2-MkP population. MPP2-MkP highlights MkP cells in the MPP2 population because MPP is a heterogeneous population. (B) Hierarchical relationship among HSCs, CMPs, MgPs, and MkPs. HSCs give rise to CMPs. CMPs give rise to MgPs. MgPs give rise to MkPs. CMP = common myeloid progenitors, HPC = hematopoietic progenitor cell, HSC = hematopoietic stem cell, MgPs = megakaryocyte lineage-committed progenitors, MkPs = megakaryocyte progenitors.

Transcriptional analyses suggested that HSCs give rise to MkPs through MgPs (**Fig. 5**). Since MgPs are widely distributed in several transcriptionally heterogeneous populations, we did not find any gene expressed specifically in MgPs. Interestingly, MgPs may be marked by the coexpression of *CD41* and *Pf4* rather than that of *CD41* and *Vwf* at the transcriptional level (**Fig. 6**). Our data together suggested that HSC1/HPC1-P2 cells give rise to HSC1/HPC1-P1/3 cells, which give rise to MkP cells in the MPP2 population (MPP2-MkP cells). These cells finally give rise to MkP cells (**Fig. 7A**). The corresponding model for a hierarchical organization of HSCs, CMPs, MgPs, and MkPs is simply shown in Figure 7B. Whether functional CMPs exist in the originally defined population is controversial.^10,13,39^ CMPs may exist in other populations.^4^ In this study, EMk-forming cells were never detected by single-cell colony assays with SCF+TPO+IL-3+EPO, suggesting that either E or Mk lineage commitment takes place independently of Mk-E progenitors. Recent human studies have suggested that lineage commitment is a continuous process.^40–42^ In our model, Mk lineage commitment occurs in a stochastic manner after a CMP stage as thought a long time ago.^43–45^ Further study is required to verify our model.

## ACKNOWLEDGMENTS

This work was supported by grants from the National Key Research and Development Program of China (2022YFA1103500, 2021YFA1100900), Science, Technology & Innovation Project of Xiongan New Area (2022XAGG0142), the National Natural Science Foundation of China (82170118, 82370116, 82250710178, 82222004, 82070112), Haihe Laboratory of Cell Ecosystem Innovation Fund (22HHXBSS00038), the Chinese Academy of Medical Sciences (CAMS) Innovation Fund for Medical Sciences (2021-I2M-1-073), Distinguished Young Scholars of Tianjin (23JCJQJC00220), and the CAMS Fundamental Research Funds for Central Research Institutes (2022-RC180-06).

## AUTHOR CONTRIBUTIONS

F.D., M.H., H.E. supervised the study; Z.L., J.W., Y.M., M.X., P.W., S.Z., X.W., F.D., H.C. performed experiments; Z.L., P.Z., M.H., H.E analyzed and interpreted data; Z.L., F.D., H.E. wrote the manuscript.

## Supplementary Material


