# Supplementary material for: Early megakaryocyte lineage-committed progenitors in adult mouse bone marrow
Source: Blood Sci. 2024 May 7;6(2):e00187. doi: 10.1097/BS9.0000000000000187 (PMC11078525; doi:10.1097/BS9.0000000000000187)
Supplement: Supplementary file 1 [file bs9-6-e00187-s001.pdf]

Supplemental Figure 1. MPP2 and MkP gating (A) The gating strategy of MPP2 cell is shown. The expression of CD34, CD41, and CD201 in MPP2 cells is additionally demonstrated. (B) The gating strategy of MkP is shown. Note the majority of $CD150^+CD48^+Flk2^-c\text{-}Kit^+Sca\text{-}1^+Lin^-$ MPP2 cell express a low level of CD34 and CD41.

A

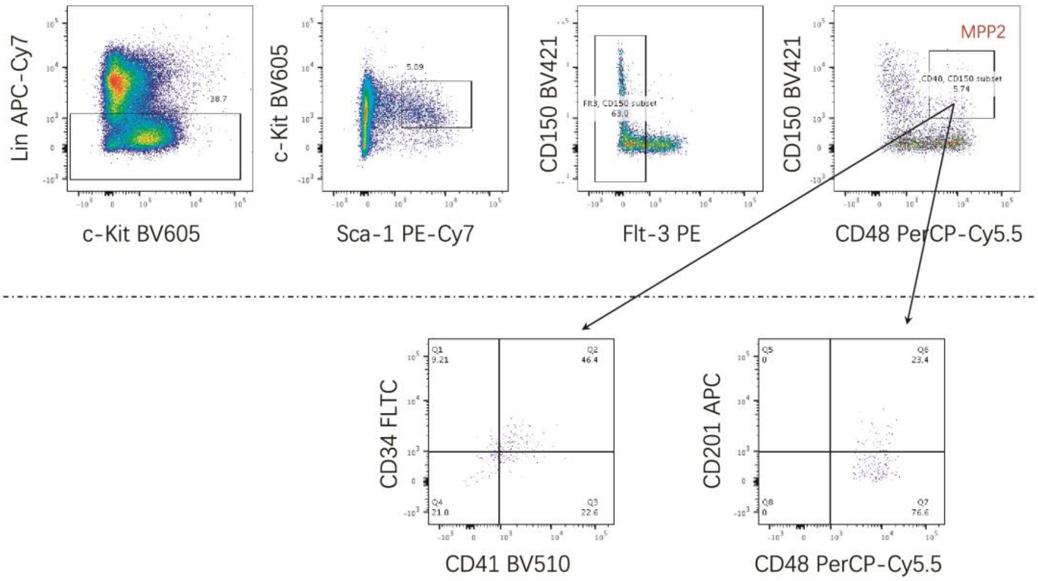
Lin APC-Cy7
c-Kit BV605
38.7
c-Kit BV605
Sca-1 PE-Cy7
5.09
CD150 BV421
Flt-3 PE
Flt3, CD150 subset
63.0
CD150 BV421
CD48 PerCP-Cy5.5
MPP2
CD48, CD150 subset
5.74
CD34 FLTC
CD41 BV510
Q1
9.21
Q2
46.4
Q4
21.8
Q3
22.6
CD201 APC
CD48 PerCP-Cy5.5
Q5
0
Q6
23.4
Q8
0
Q7
76.6

B

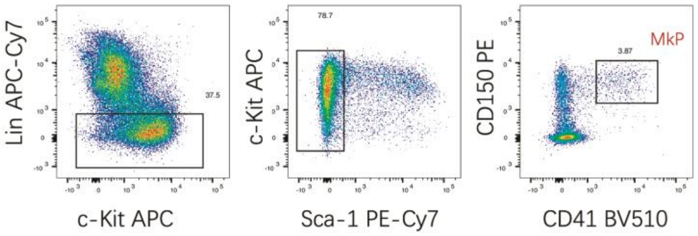
Lin APC-Cy7
c-Kit APC
37.5
c-Kit APC
Sca-1 PE-Cy7
78.7
CD150 PE
CD41 BV510
3.87
MkP
