# Supplementary material for: Early megakaryocyte lineage-committed progenitors in adult mouse bone marrow
Source: Blood Sci. 2024 May 7;6(2):e00187. doi: 10.1097/BS9.0000000000000187 (PMC11078525; doi:10.1097/BS9.0000000000000187)
Supplement: Supplementary file 2 [file bs9-6-e00187-s002.pdf]

Supplemental Table 1. Gene set for scRT-PCR.

Probes	Genes	Probes	Genes
Mm00443316_m1	*CD150*	Mm00455932_m1	*CD48*
Mm00455932_m1	*c-Kit*	Mm00726565_s1	*Ly6a*
Mm00518378_m1	*Esam*	Mm00440992_m1	*Procr*
Mm00522487_m1	*Pdzk1IP*	Mm00515855_m1	*Gfi-1*
Mm00492318_m1	*Gfi-1b*	Mm00841873_m1	*Ly6c2*
Mm04934123_m1	*Ly6g*	Mm00514283_s1	*CEBPa*
Mm00843434_s1	*CEBPb*	Mm02030363_s1	*CEBPe*
Mm00514283_s1	*Spib*	Csf1r/CD115	*Csf1r*
Mm00432735_m1	*Csf2ra*	Mm00432735_m1	*Csf3r*
Mm00801807_m1	*CD11a*	Mm00434455_m1	*CD11b*
Mm00498698_m1	*CD11c*	Mm00438094_g1	*CD14*
Mm00438874_m1	*CD64*	Mm00522487_m1	*CD16*
Mm00492301_m1	*GATA1*	Mm00492301_m1	*GATA2*
Mm00484683_m1	*GATA3*	Mm00494336_m1	*Zfpm1*
Mm04208330_g1	*KLF1*	Mm00500486_g1	*KLF2*
Mm00522487_m1	*CD71*	Mm00833882_ml	*EPOR*
Mm00801891_m1	*NFE2*	Mm04208330_g1	*TAL1*
Mm00440156_m1	*Ldb1*	Mm00801891_m1	*ALAS2*
Mm00443980_m1	*PF4*	Mm00550376_m1	*Vwf*
Mm00439741_m1	*CD41*	Mm00438874_m1	*Gp9/CD42*
Mm00443980_m1	*CD61*	Mm00440310_m1	*Mpl*
Mm01318991_m1	*Mef2a*	Mm01340842_m1	*Mef2c*
Mm00439016_m1	*Fli1*	Mm00439016_m1	*FLT3*
Mm00607939_s1	*GAPDH*	Mm00607939_s1	*Actb*

Forty-eight genes were selected for scRT-PCR.

Supplemental Table 2. Myeloid lineage commitment in early divisions of HSCs.

Cytokines	Cells /well	No. of colonies								No. of clones
		nmEMk	nmE	nmMk	nm	n	m	Mk	U	
TPO	3	3	0	0	0	0	0	0	0	1
	4	4	0	0	0	0	0	0	0	3
		3	0	0	1	0	0	0	0	3
		3	0	0	0	0	1	0	0	1
		2	0	0	2	0	0	0	0	3
		2	0	0	1	0	1	0	0	1
		1	1	0	0	0	2	0	0	1
		1	0	0	1	0	2	0	0	1
	5	5	0	0	0	0	0	0	0	1
	6	4	1	1	0	0	0	0	0	1
		1	0	0	2	0	2	0	1	1
	7	7	0	0	0	0	0	0	0	1
		5	1	0	0	0	0	0	1	1
		2	1	0	1	0	0	0	3	1
	8	6	0	0	0	0	0	2	0	1
		2	0	0	5	0	1	0	0	1
		2	0	0	2	0	4	0	0	1
		1	0	0	3	0	4	0	0	1
SCF + TPO	4	4	0	0	0	0	0	0	0	1
		3	1	0	0	0	0	0	0	1
		2	1	0	1	0	0	0	0	2
		1	0	2	1	0	0	0	0	1
		1	0	1	1	0	1	0	0	1
		3	0	0	0	0	0	0	1	1
		2	0	0	1	0	0	0	1	1
		1	0	1	0	0	1	0	1	1
	5	4	0	0	0	0	1	0	0	1
		3	0	0	2	0	0	0	0	1
		1	1	2	0	0	1	0	0	1
	6	5	0	0	1	0	0	0	0	1
		5	0	0	0	0	0	0	1	1
		1	0	0	3	0	0	0	2	1
	7	7	0	0	0	0	0	0	0	1
		2	1	1	1	0	0	0	2	1
	8	5	0	0	1	0	0	0	3	1
		2	0	0	0	0	3	0	3	1
Total	224	126	9	8	37	0	24	2	19	43

Single HSC1-P2 cells were cultured in serum-free medium in the presence of 50 ng/ml TPO for 4 days or in the presence of 50 ng/ml SCF + 50 ng/ml TPO for 3 days as described [25]. All cells in a well were individually transferred into wells with the medium of single-cell colony assay by micromanipulator. Cells were further incubated for 14 days. Colony cells were morphologically identified as neutrophils (n), macrophages (m), erythroblasts (E), and megakaryocytes (Mk). When single cells were lost during transfer or when colony cells were lost during Cytospin preparation, colonies were classified as unidentified colonies (U).

Supplemental Table 3. Comparison of cell surface makers among HSC, MgP, and MkP populations

Cells	HSCs/CMPs		MgPs		MkP cells	
Populations	HSC1 P2	HPC1 P2	HSC1/HPC1 P1	HSC1/HPC1 P3	MPP2 (Passegue)	MkP (Bryder)
CD201	+	+	-	-/+	-/+	-
CD150	+	+	+	+	+	+
CD48	-	-	-	+	+	+
CD41	-	+	-/+	-/+	+	+
CD34	-	-	-	-	+*	+
c-Kit	+	+	+	+	+	+
Sca-1	+	+	+	+	+	-
Lineage	-	-	-	-	-	-
CD135	-	-	-	-	-	-

"MgPs and MkP cells" were used as the name of functional megakaryocyte progenitors whereas "MkP" was used as the name of phenotypically defined megakaryocyte progenitor population in this study. *, MPP2 has been originally defined as a CD34-positive population. But, the CD34 marker was not used in Passegue's study. The HPC1-P3 population may overlap the CD34-negative MPP2 population when CD34 is not used for MPP2 (see Supplemental Fig. 1). -/+, both negative and positive cells are detectable.
