# Supplementary material for: Early megakaryocyte lineage-committed progenitors in adult mouse bone marrow
Source: Blood Sci. 2024 May 7;6(2):e00187. doi: 10.1097/BS9.0000000000000187 (PMC11078525; doi:10.1097/BS9.0000000000000187)
Supplement: Supplementary file 3 [file bs9-6-e00187-s003.pdf]

Supplemental Figure 2. Morphological analysis of colony cells. (A) Morphology of colony cells. Mk, megakaryocytes; n, neutrophils; m, macrophages; E, erythroblasts. (B) Different types of colonies: nmEMk, nmMk, nm, and m colonies are shown. Cells were collected from a well of 96-plates and spin down onto glass slides, and subjected to Wright Giemsa staining.

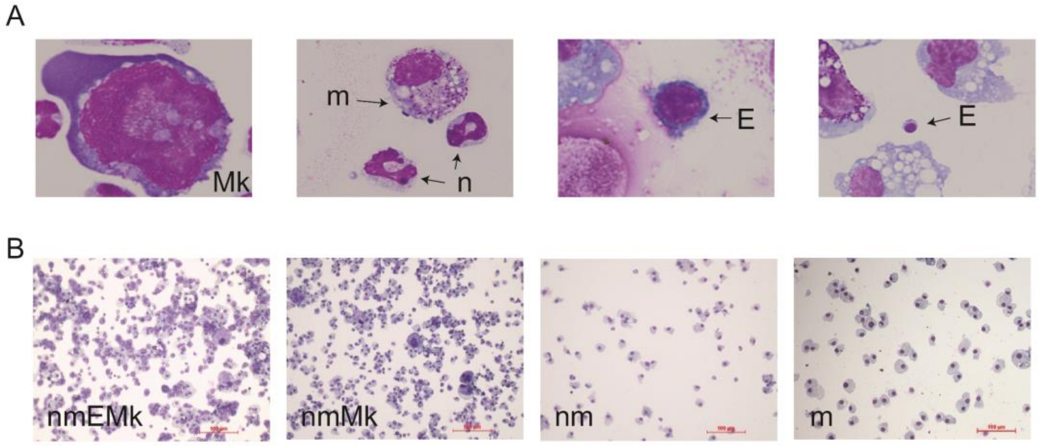
A
Mk
m
n
n
E
E
B
nmEMk
nmMk
nm
m
