# Supplementary material for: Early megakaryocyte lineage-committed progenitors in adult mouse bone marrow
Source: Blood Sci. 2024 May 7;6(2):e00187. doi: 10.1097/BS9.0000000000000187 (PMC11078525; doi:10.1097/BS9.0000000000000187)
Supplement: Supplementary file 4 [file bs9-6-e00187-s004.pdf]

Supplemental Figure 3. Additional colony assays. (A) Single-cell colony assay for MPP2 and MkP. Sixty single cells were sorted and cultured for 14 days (n=3). The colony-forming efficiency of $CD150^+CD48^+Flk2^-c\text{-}Kit^+Sca\text{-}1^+Lin^-$ MPP2 cells was 52.2 ± 0.3 % (left panel). Most $CD150^+CD41^+c\text{-}Kit^+Sca\text{-}1^-Lin^-$ MkP cells (98.9%) only gave rise to Mks (right panel). (B) Thirty single cells from the HSC1/HSC2/HPC1-P1/2/3 populations were cultured for 21 days in two independent experiments. On day 21, colonies were scored, and cell components were identified. The frequency of nmEMk colony-forming cells in HSC1-P2 cells was significantly greater than that in HSC1-P1 or -P3 cells (71.1% versus 29.6% or 30.6%, $p < 0.001$). The frequency of nmEMk colony-forming cells in HSC2-P2 cells was significantly greater than that in HSC2-P3 cells (39.1% versus 14.3%, $p < 0.001$). The frequency of nmEMk colony-forming cells in HPC1-P2 cells was significantly greater than that in HPC1-P1 or -P3 cells (58.6% versus 13.8% or 7.1%, $p < 0.001$). The frequency of Mk colony-forming cells in HSC1-P1 or -P3 was significantly greater than that in HSC1-P2 cells (40.7% or 50.0% versus 4.4%, $p < 0.001$). The frequency of Mk colony-forming cells in HPC1-P1 or -P3 cells was significantly greater than that in HPC1-P2 cells (65.5% or 78.6% versus 24.1%, $p < 0.001$). Statistical analysis was performed by chi-square test.

A

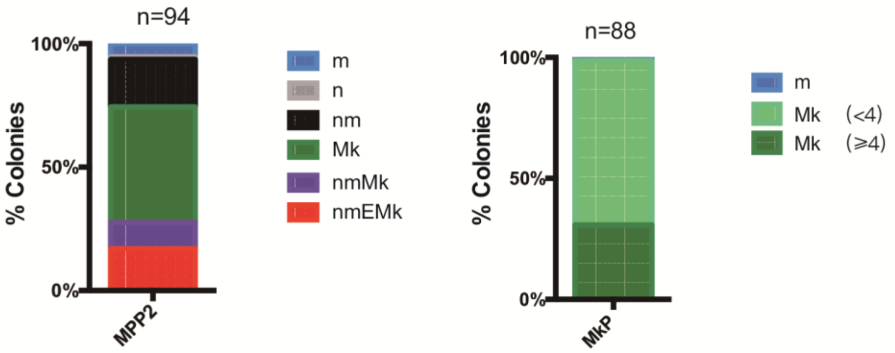
n=94
100%
50%
0%
% Colonies
MPP2
m
n
nm
Mk
nmMk
nmEMk
n=88
100%
50%
0%
% Colonies
MkP
m
Mk (<4)
Mk (≥4)

B

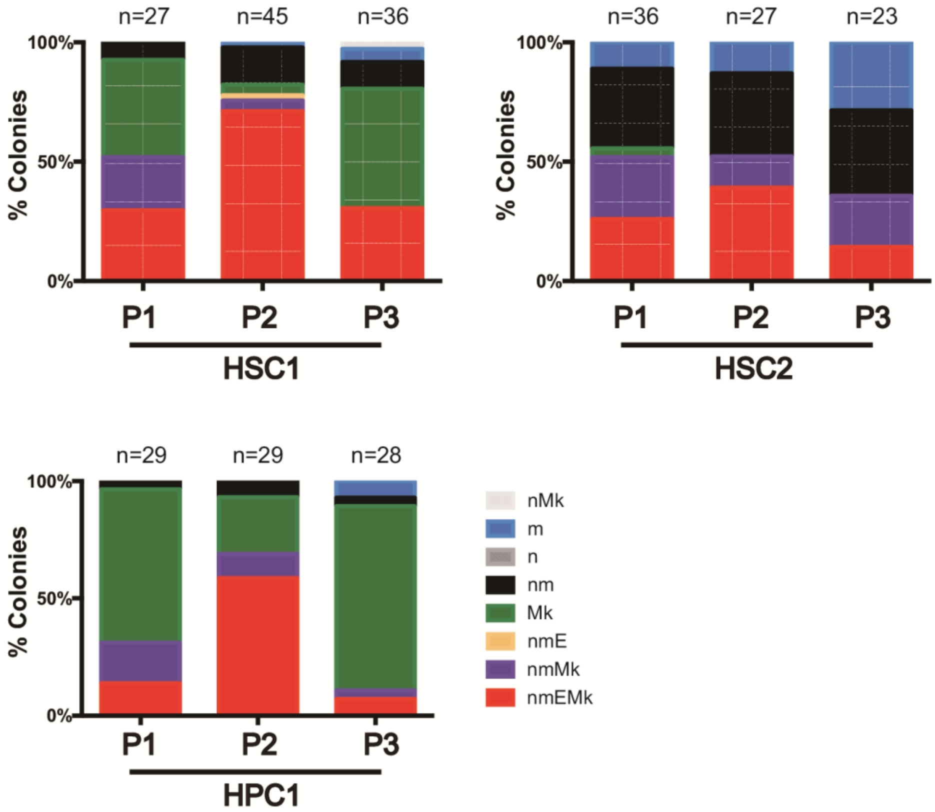
n=27
n=45
n=36
100%
50%
0%
% Colonies
P1
P2
P3
HSC1
n=36
n=27
n=23
100%
50%
0%
% Colonies
P1
P2
P3
HSC2
n=29
n=29
n=28
100%
50%
0%
% Colonies
P1
P2
P3
HPC1
nMk
m
n
nm
Mk
nmE
nmMk
nmEMk
