# Supplementary material for: Early megakaryocyte lineage-committed progenitors in adult mouse bone marrow
Source: Blood Sci. 2024 May 7;6(2):e00187. doi: 10.1097/BS9.0000000000000187 (PMC11078525; doi:10.1097/BS9.0000000000000187)
Supplement: Supplementary file 5 [file bs9-6-e00187-s005.pdf]

Supplemental Figure 4. Long-term reconstitution by 10 cells.

Ten cells each from the HSC1, HSC2 and HPC1 populations along with $5x10^5$ competitor cells were injected into 10 lethally irradiated mice. Peripheral blood was examined 1, 3, and 6 months after transplantation. Secondary transplantation was performed 6 months after transplantation.

(A) Results of transplantation with 10 HSC1 cells. All mice survived. Hematopoietic reconstitution was observed in 5 of 10 recipients of HSC1-P1 cells after primary transplantation but in none of the recipients after secondary transplantation (Supplemental Fig. 4A). Reconstitution was observed in 8 of 10 recipients of HSC1-P2 cells after primary transplantation and in 8 of 10 recipients after secondary transplantation. Reconstitution was observed in only 1 of 10 recipients of HSC1-P3 cells after primary transplantation, the level of which decreased by 6 months and became undetectable after secondary transplantation.

(B) Results of transplantation with 10 HSC2 cells. All mice survived. Reconstitution was observed in 4 of 10 recipients of HSC2-P1 cells after primary transplantation but not after secondary transplantation. Reconstitution was observed in 9 of 10 recipients of HSC2-P2 cells after primary transplantation and in 2 of 10 recipients after secondary transplantation, but its level gradually decreased. No reconstitution was observed in recipients of HSC2-P3 cells after primary and secondary transplantation.

(C) Results of transplantation with 10 HPC1 cells. Seven recipients of HPC1-P1 cells, 9 recipients of HPC1-P2 cells, and 7 recipients of HPC1-P3 cells survived. No reconstitution was observed in recipients of HPC1-P1 cells after primary and secondary transplantation. Reconstitution was observed in 5 of 10 recipients of HPC1-P2 cells after primary transplantation but not after secondary recipients. Reconstitution was observed in 1 of 10 recipients of HPC1-P3 cells after primary transplantation but not after secondary transplantation. The reconstitution level of HPC1-P2 cells was significantly lower and decreased faster than that of HSC1-P2 cells.

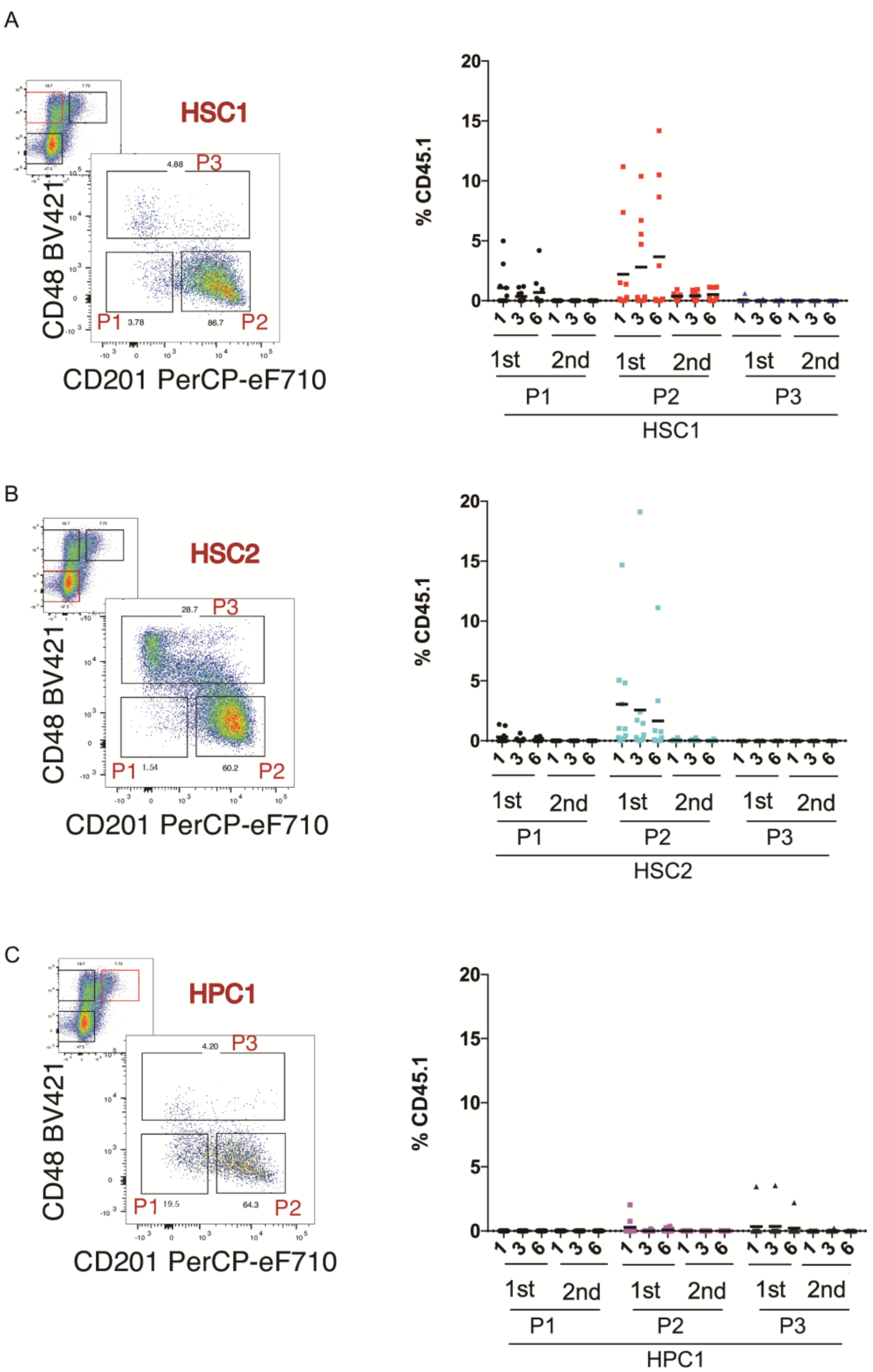
A
HSC1
CD48 BV421
CD201 PerCP-eF710
P1 3.78
P2 86.7
P3 4.88
% CD45.1
1st
2nd
P1
P2
P3
HSC1
B
HSC2
CD48 BV421
CD201 PerCP-eF710
P1 1.54
P2 60.2
P3 28.7
% CD45.1
1st
2nd
P1
P2
P3
HSC2
C
HPC1
CD48 BV421
CD201 PerCP-eF710
P1 19.5
P2 64.3
P3 4.20
% CD45.1
1st
2nd
P1
P2
P3
HPC1
