# Supplementary material for: Early megakaryocyte lineage-committed progenitors in adult mouse bone marrow
Source: Blood Sci. 2024 May 7;6(2):e00187. doi: 10.1097/BS9.0000000000000187 (PMC11078525; doi:10.1097/BS9.0000000000000187)
Supplement: Supplementary file 6 [file bs9-6-e00187-s006.pdf]

Supplemental Figure 5. Gene expression analysis based on scRNA-seq data. (A-D) The expression of Mk-related genes (*Vwf*, *Pl4*, *Gata1*, and *Runx1*) and **(E-H)** the expression of HSC-related genes (*Procr*, *Sh2b3*, *Mecom*, and *Spi1*) are selectively shown after a standard log normalization step. *, $p<0.05$; **, $p<0.01$; ***, $p<0.001$; ****, $p<0.0001$ (Wilcoxon test).

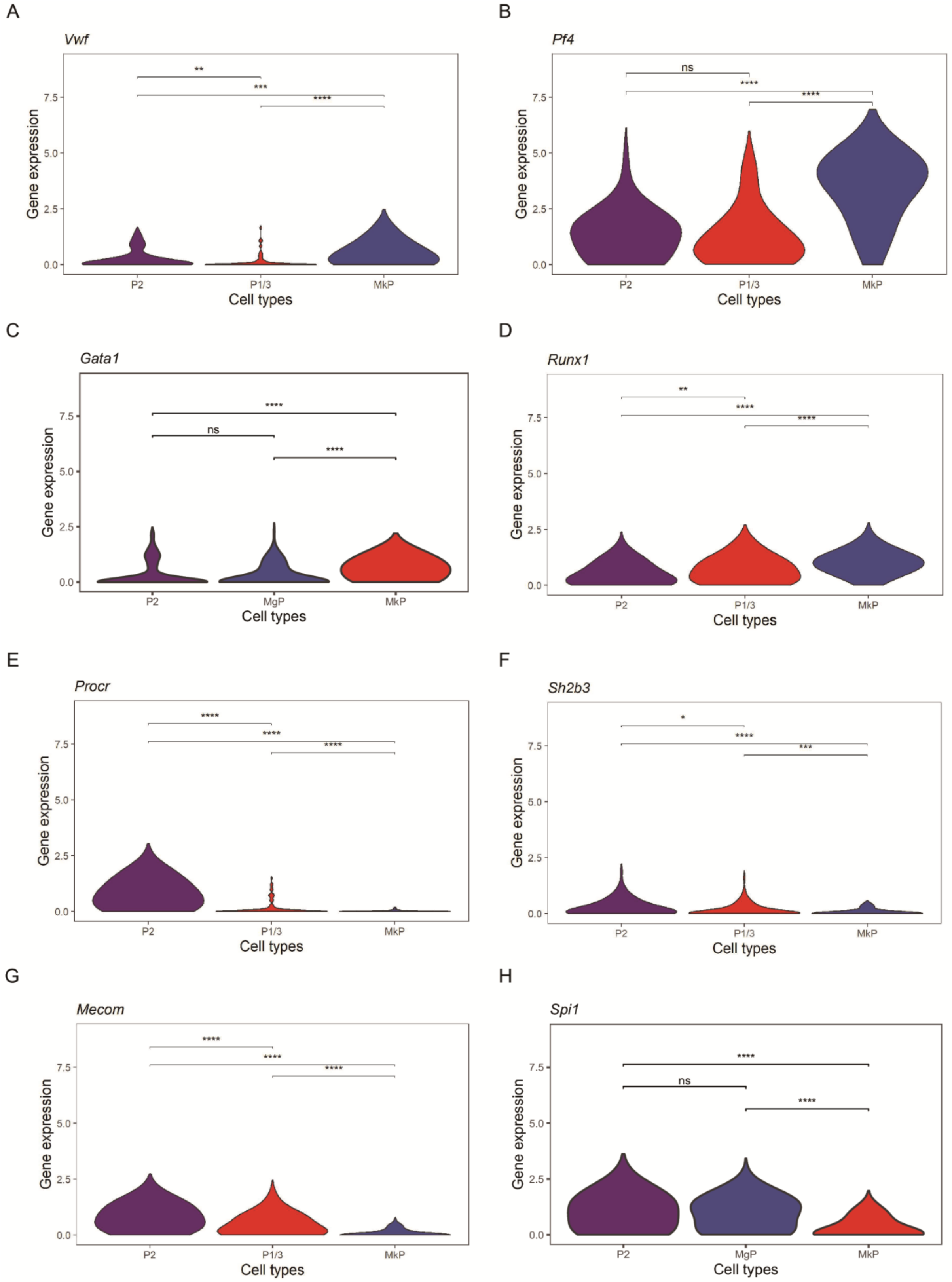

A
Vwf
**
***
****
Gene expression
Cell types
P2
P1/3
MkP
B
Pf4
ns
****
****
C
Gata1
****
ns
****
P2
MgP
MkP
D
Runx1
**
****
****
E
Procr
****
****
****
F
Sh2b3
*
****
***
G
Mecom
****
****
****
H
Spi1
****
ns
****
