# Supplementary material for: Early megakaryocyte lineage-committed progenitors in adult mouse bone marrow
Source: Blood Sci. 2024 May 7;6(2):e00187. doi: 10.1097/BS9.0000000000000187 (PMC11078525; doi:10.1097/BS9.0000000000000187)

Supplemental Figure 6. Single-cell RT-PCR analysis of HSC1/HSC2/HPC1-P1/2/3.

The heatmaps with 48 genes in the horizontal rows and 24 single cells in the vertical columns. Positive cells are defined as the threshold cycle values [Ct] < 27.65.

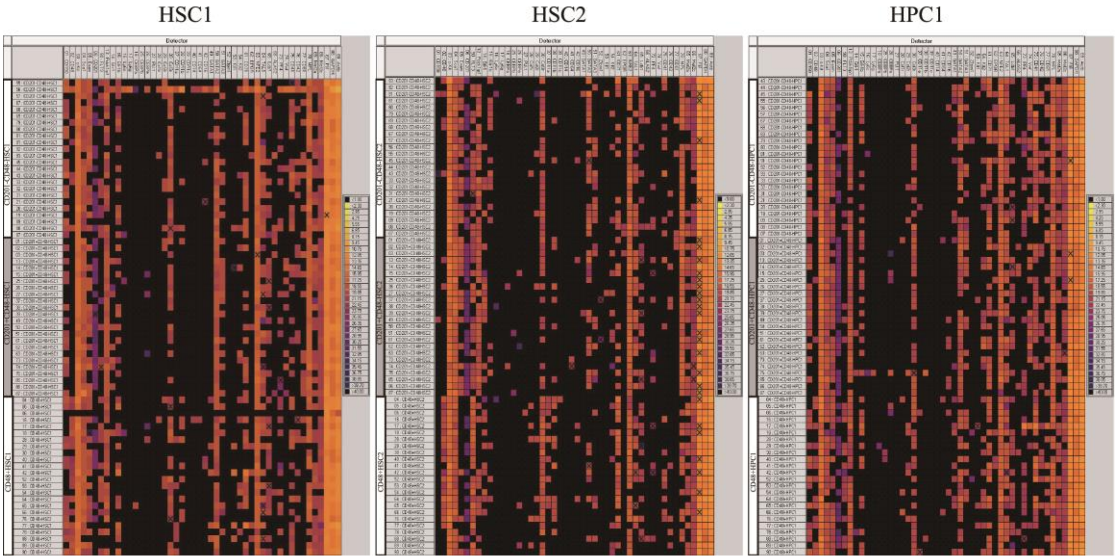

Supplement: Supplementary file 7 [file bs9-6-e00187-s007.pdf]
